# CRISPR/Cas9 Genome Editing in LGMD2A/R1 Patient-Derived Induced Pluripotent Stem and Skeletal Muscle Progenitor Cells

**DOI:** 10.1155/2023/9246825

**Published:** 2023-11-09

**Authors:** Lampros Mavrommatis, Abdul Zaben, Urs Kindler, Marie-Cécile Kienitz, Julienne Dietz, Hyun-Woo Jeong, Pierre Böhme, Beate Brand-Saberi, Matthias Vorgerd, Holm Zaehres

**Affiliations:** ^1^Ruhr University Bochum, Medical Faculty, Institute of Anatomy, Department of Anatomy and Molecular Embryology, 44801 Bochum, Germany; ^2^Ruhr University Bochum, Medical Faculty, Department of Neurology with Heimer Institute for Muscle Research, University Hospital Bergmannsheil, 44789 Bochum, Germany; ^3^Max Planck Institute for Molecular Biomedicine, Department of Cell and Developmental Biology, 48149 Münster, Germany; ^4^Ruhr University Bochum, Medical Faculty, Department of Cellular Physiology, 44801 Bochum, Germany; ^5^Witten/Herdecke University, Institute of Virology and Microbiology, Department of Human Medicine, Faculty of Health, 58453 Witten, Germany; ^6^Max Planck Institute for Molecular Biomedicine, Sequencing Core Facility, 48149 Münster, Germany; ^7^Ruhr University Bochum, Department of Psychiatry, Psychotherapy and Preventive Medicine, LWL University Hospital Bochum, 44791 Bochum, Germany

## Abstract

Large numbers of Calpain 3 (CAPN3) mutations cause recessive forms of limb-girdle muscular dystrophy (LGMD2A/LGMDR1) with selective atrophy of the proximal limb muscles. We have generated induced pluripotent stem cells (iPSC) from a patient with two mutations in exon 3 and exon 4 at the calpain 3 locus (W130C, 550delA). Two different strategies to rescue these mutations are devised: (i) on the level of LGMD2A-iPSC, we combined CRISPR/Cas9 genome targeting with a FACS and Tet transactivator-based biallelic selection strategy, which resulted in a new functional chimeric exon 3-4 without the two CAPN3 mutations. (ii) On the level of LGMD2A-iPSC-derived CD82+/Pax7+ myogenic progenitor cells, we demonstrate CRISPR/Cas9 mediated rescue of the highly prevalent exon 4 CAPN3 mutation. The first strategy specifically provides isogenic LGMD2A corrected iPSC for disease modelling, and the second strategy can be further elaborated for potential translational approaches.

## 1. Introduction

Muscular dystrophies compose a heterogeneous group of more than thirty skeletal muscle genetic disorders, which are caused by genetic alterations in proteins located in the extracellular matrix, plasma/nuclear membrane, and sarcomere/cytoplasm of skeletal muscle cells. Despite their heterogenicity, the dystrophic phenotype is characterized by an initial muscle weakening that progresses and, overtime, concludes into complete disarray of skeletal muscle organization, unable to support movement. Classification with respect to predominant skeletal muscle weakness distribution delineates muscular dystrophies into six major forms [1]. One of these, limb-girdle muscular dystrophies (LGMDs) embraces more than thirty subtypes and clinically presents with selective atrophy of the proximal limb muscles. Depending on the way of inheritance, they are classified as LGMD type 1 (dominant) and LGMD type 2 (recessive). Despite the variability on age onset and symptom severity among different subtypes, the dystrophic phenotype affects symmetrically the proximal limb girdle musculature. The initial weakness slowly progresses and over time concludes into muscle wasting that eventually restricts body movements and causes alterations in posture. In addition, in more severe subtypes, cardiac and respiratory muscles are affected.

Limb-girdle muscular dystrophy type 2A (LGMD2A), a subtype with higher prevalence among LGMDs, does not arise from a defect of the structural cellular composition, but rather from a defect of a skeletal muscle-specific protease, the Calpain 3 (CAPN3) [2–4]. The Calpain 3 gene, in humans, is located at the long arm of chromosome 15 (15q15.1-q21.1). The CAPN3 locus is composed of 24 exons that extend over a genomic region of 53 kb and lead to a transcript of 3.5 kb. To date, over 280 pathogenic mutations have been documented that span the entire sequence. Among them, most common are missense mutations in domains II and III that affect directly the enzyme activity in domain II or indirectly catalytic triad formation at the active site [5]. In addition, several pathogenic missense mutations disrupt the Calpain 3-titin binding [6, 7]. These missense mutations affect the Calpain 3 stability, as it is unstable in its free form due to its high autocatalytic activity [8].

Calpain 3, a calcium-dependent 94 kDa enzyme, belongs to the group of soluble cysteine proteases and consists of four protein domains [9]: the proline peptide domain I, the protease catalytic center II, the calcium and phospholipid binding domain III, and the calmodulin-like Ca2+ binding domain IV. CAPN3 exhibits skeletal muscle tissue expression with at least ten-fold higher expression in muscle than other lineages [10]. Despite the homology of CAPN3 in respect to the large subunit of conventional calpains (50%), it has significant variations in its amino acid sequence [11]. In general, the function of CAPN3 is still unknown, but there are hints from various experiments for its contribution in biological processes like apoptosis, muscle cell differentiation and remodeling, sarcomere formation, and membrane repair. Baghdiguian et al. [12] reported that CAPN3 deficiency leads to myonuclear apoptosis through profound perturbation of the IkappaB alpha/NF-kappaB pathway. Yeast two-hybrid mapping assays demonstrated that CAPN3 binds to two regions of titin positioning CAPN3 in processes like regulation of cytoskeleton and sarcomere remodeling [13].

Biopsies from patients with LGMB2B or Miyoshi myopathy (autosomal recessive muscle diseases caused by dysferlin mutations) and results from in vivo mouse studies propose as additional CAPN3 substrates, membrane proteins, or proteins involved in membrane repair pathways [14]. Coimmunoprecipitation assays prove that CAPN3 and dysferlin interact biochemically resulting in CAPN3 stabilization via its anchorage to the plasma membrane [15, 16]. CAPN3-deficient mouse models present with compromised membrane integrity [17].

Besides all these approaches in cell culture and mice, patient biopsies are difficult to procure in sufficient amounts to study calpain-3 function in human in vitro systems in more detail. The current state of research allows reprogramming of patient cells into pluripotent stem cells [18], generation of isogenic lines by genome editing techniques [19], and differentiation of pluripotent lines to various cell lineages, which carry the genetic mutations of patients [20].

**Figure 1:**
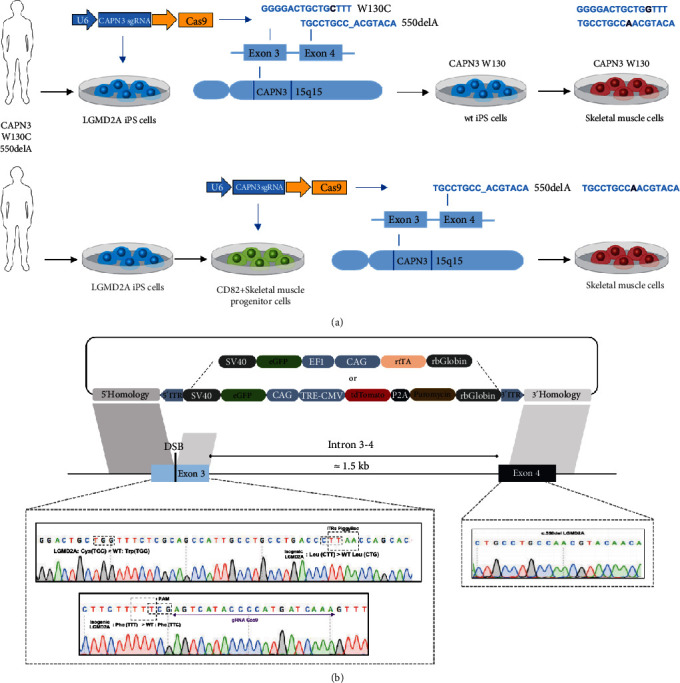
(a) Induced pluripotent stem cells and genome editing to model genetic correction of limb-girdle muscular dystrophy (LGMD2A). CRISPR/Cas9 mediated homology-directed repair (HDR) at the calpain-3 (CAPN3) locus on the level of human LGMD2A iPS cells and subsequent differentiation to wildtype (wt) skeletal muscle cells (upper panel). CRISPR/Cas9 mediated HDR at the CAPN3 locus on the level of human iPS-derived CD82+ skeletal muscle progenitor cells (lower panel). (b) Strategy to perform gene correction of LGMD 2A patient hiPS cell line: cartoon illustrating the targeting strategy followed for generating the isogenic line using CRISPR/Cas9 genome editing.

In our study, we present reprogramming of a LGMD2A patient's fibroblasts to induced pluripotent stem cells (iPSC) and generation of an isogenic control line by rescuing the patient's both mutations with CRISPR/Cas9 genome editing on the level of iPSC (Figure 1(a)). In a second line of experiments, we differentiate CD82+ skeletal muscle progenitor cells from these LGMD2A iPSC and demonstrate CRISPR/Cas9 mediated rescue of the exon 4 CAPN3 mutation in these progenitor cells. We, thereby, offer a direct comparison between a LGMD2A dystrophic line and its isogenic form, an approach that can provide further evidence of CAPN3 mechanisms of action.

## 2. Materials and Methods

### 2.1. Human-Induced Pluripotent Stem Cell (iPSC) Culture

Human-induced pluripotent stem cell (hiPSC) lines, Cord Blood iPSC (CB CD34+, passage 15–35) [21], LGMD2A patient iPSC (passage 5-25), and isogenic LGMD2A Exons 3-4 edited (passage 2-13) were cultured in TESR-E8 (StemCell Technologies) on Matrigel GFR- (Corning-) coated 6 well plates.

### 2.2. Human iPSC Generation

The LGMD2A/R1 patient material was collected at the University Hospital Bergmannsheil after ethical approval from the ethics commission of the Ruhr-University Bochum, Medical Faculty (15-5401, 08/2015). Human dermal fibroblasts (HDFs) were taken as a biopsy from the LGMD2A patient and grown in Dulbecco's modified Eagle's medium with high glucose containing 10% fetal bovine serum, penicillin, and streptomycin. A polycistronic lentiviral vector encoding the human cDNAs of OCT4, SOX2, KLF4, and c-MYC (OSKM) and dtTomato under the control of the SFFV promoter was produced as previously described [21–23]. The HDFs were seeded at 1 × 10^5^ cells per well of a 6-well plate. On the next day, the cells were transduced with concentrated OSKM lentivirus. Six days after transduction, fibroblasts were harvested by trypsinization and replated at 8 × 10^4^ cells per well of a 6-well plate on a mitomycin C-treated STO feeder layer (Sigma-Aldrich, USA). On the following day, the medium was replaced with DMEM/F12 (Invitrogen, USA) supplemented with 20% knockout serum replacement (Invitrogen, USA), 2 mM L-glutamine (Invitrogen, USA), 1% nonessential amino acids (Invitrogen, USA), 0.1 mM *β*-mercaptoethanol (Invitrogen, USA), 1% penicillin/streptomycin, and 10 ng/ml bFGF (R&D). 50 *μ*g/ml of vitamin C (Sigma-Aldrich, USA) and 0.5 mM valproic acid (VPA, Sigma-Aldrich, USA) were added till the appearance of iPSC-like cells. The medium was changed every day. Colonies were picked up mechanically and transferred into mitomycin C-treated STO feeder layers.

### 2.3. Human iPSC Characterization

#### 2.3.1. Immunohistochemistry for Pluripotency Markers

For immunocytochemical staining of the pluripotency markers, iPSCs were fixed with 4% paraformaldehyde for 15 min at room temperature. The cells were permeabilized with 0.1% Triton X-100 and then blocked with 1% bovine serum albumin (Amresco, Inc., USA). Staining was carried out using primary anti-Oct4 (1:  200, Santa Cruz, USA), anti-Sox2 (1:  200, ThermoFisher Scientific, USA), anti-SSEA4 (1:  200, Chemicon, MA, USA), and anti-Tra-1-60 (1:  200, Chemicon, MA, USA).

#### 2.3.2. Expression Profiling

Total RNA was extracted from iPSC lines (LGMD2A, CB-CD34+) using the RNAeasy Micro Kit (Qiagen) according to the manufacturer's instructions. The RNA integrity was evaluated on an Agilent 2100 Bioanalyzer by using the RNA 6000 Pico kit (Agilent). The cDNA library was prepared by using the whole transcriptome Illumina TruSeq Stranded Total RNA Library Prep Kit Gold (Illumina), followed by evaluation on an Agilent 2100 Bioanalyzer by using the DNA 1000 kit. The resulting mRNA library was sequenced as 2 × 75 bp paired-end reads on a NextSeq 500 sequencer (Illumina). Sequenced reads were aligned to the human reference genome (hg38) with TopHat2 (version 2.1.1), and the aligned reads were used to quantify mRNA expression by using HTSeq-count (version 0.11.2). iPSCs were compared to already available transcriptomic datasets of the human embryonic stem cell lines H1 and H9.

#### 2.3.3. Microarray Karyotype Analysis

Genomic DNA from the patient and genome-edited (isogenic) LGMD2A pluripotent stem cell lines were purified using DNeasy blood and tissue kit (Qiagen). Samples were further processed at the University of Bonn Life & Brain Genomics Facility using Illumina iScan technology (https://www.lifeandbrain.com/produkte-services/lifeandbrain-genomics/).

### 2.4. CRISPR/Cas9 Genome Editing of LGMD2A iPSC

#### 2.4.1. Biallelic-Selection Strategy at CAPN3 Exon3 and Exon4 on the Level of iPSC

The selection cassette system was incorporated into plasmids flanked by 1 kb in length homology arms of the Calpain 3 locus. The upper homology arm sequence was corresponding to the wild type sequence of exon 3 {130Trp(TGG) > Cys(TGC)} of the Calpain 3 locus till the point where by a silent mutation (CTG > CTT), a TTAA sequence corresponding to the inverted terminal repeats (ITRs) of the PiggyBac system could be generated. This TTAA sequence marked the start for the selection cassette. The selection cassette system itself composed of two plasmids, one expressing the reverse tetracycline activator (rtTA) protein under the constitutively active CAG promoter and the other expressing tdTomato plus puromycin another under the inducible TRE-CMV promoter. This approach generates a depending loop between the expression cassettes, ensuring that only upon integration of both plasmids red fluorescence can be detected. In addition, to ensure that both alleles are “selection cassettes free” upon PiggyBac mediated excision removal, it was a necessity that both plasmids were expressing the eGFP protein under a constitutively active promoter. The sequence downstream, the inverted terminal repeats (ITRs) sequence of the PiggyBac system (TTAA), corresponded to the lower homology arm of the selection system. To generate the new chimeric exon 3/4 exon without introducing any further alterations, the sequence of the lower homology arm was a chimera between the rest of the exon 3 sequence followed directly by the EXON4 wild type sequence, while skipping the sequence of the intermediate intron. Further, to avoid alterations on the selection cassette introduced by CAS9 nuclease, the PAM sequence of the cassette was mutated by introducing another silent mutation (TTC > TTT). The gRNA sequence introduced into the Cas9 plasmid (Addgene Plasmid #58766) was 5′-TTT.GAT.CAT.GGG.GTA.TGA.CT-3′. For performing genome editing of the CAPN3 locus at exon 3 and exon 4 sites, constructs containing selection cassettes together with the Cas9 nuclease were delivered to the cells using lipofectamine stem cell transfection reagent (ThermoFisher Scientific). After transfection, the edited population was enriched first by puromycin (Sigma-Aldrich) selection (0.5 ug/ml) upon doxycycline administration at a concentration of 1 ug/ml followed by sorting for dtTomato-eGFP positive cells (BD Biosciences AriaII). At this stage, the genomic integrity of the CAPN3 locus and the presence of the ITRs of the PiggyBac system on the genome were evaluated by PCR amplification of the edited areas using GXL Polymerase (Clontech). For the excision of the cassette, cells were transfected with constructs carrying the excision-only mutant (R372A/K375A) of the hyperactive PiggyBac transposase [24, 25] by using lipofectamine stem cell transfection reagent. After transfection, the negative fluorescent cells were selected by FACS sorting followed by genotyping for final validation. Primers used for amplification of the exon3/4 region of the calpain-3 gene were Exon3_FWD: TTCTCCTTCCCTGGGTTGAC and Exon4_REV: CTCGGCTGGATTTTGCAACC.

#### 2.4.2. Genome Editing at CAPN3 Exon 3 and 4 on the Level of Myogenic Progenitor Cells

The Cas9 and single guide RNA (sgRNA) harboring vector pX260 (Addgene plasmid #42229, [26, 27]) was used to edit the W130C and the 550delA CAPN3 mutations on chromosome 15 (15q15.1) in LGMD2A iPS cell-derived skeletal muscle progenitor cells. sgRNAs were designed to target exons 3 and 4 of CAPN3 using the Target Finder Software (Feng Zhang Laboratory, MIT, Cambridge, USA, http://crispr.mit.edu.):

CAPN3 Exon3: 5′-TCG.ATG.AAA.CTT.TGA.TCA.TGG.GGT.ATG.ACT-3′; CAPN3 Exon4: 5′-GAC.TCT.GTG.CGT.GAC.GCT.TCT.GTG.CAG.TTC-3′.

Annealed oligos (CAPN3 Exon3 5′AAACTCGATGAAACTTTGATCATGGGGTATGACTGT3′+5′TAAAACAGTCATACCCCATGATCAAAGTTTCATCGA3′ or CAPN Exon4 5′AAACGACTCTGTGCGTGACGCTTCTGTGCAGTTCGT3′+5′TAAAACGAACTGCACAGAAGCGTCACGCACAGAGTC3′) were ligated into pX260. A 156-nt single-stranded DNA oligonucleotide (ssODN) containing the wildtype nucleotide sequence (Exon3: TCTGCCTGCAGGGGACTGCTGGTTTCTCGCAGCCATTGCCTGCCTGACCCTGAACCAGCACCTTCTTTTCCGAGTCATACCCCATGATCAAAGTTTCATCGAAAACTACGCAGGGATCTTCCACTTCCAGGTGAGGTAATGAGAGTGTAG; Exon4: GAGGAATGTGGAGGAAGGACACATTTCCTAACAGTAATTTGAGTATGTGACTCTGTGCGTGACGCTTCTGTGCAGTTCTGGCGCTATGGAGAGTGGGTGGACGTGGTTATAGATGACTGCCTGCCAACGTACAACAATCAACTGGTTTTC) was used as template for homology-directed repair (HDR). The vector (1.5 *μ*g of pX260- CAPN_exon3 or 1.5 *μ*g of pX260- CAPN_exon4) and the ssODN (1.5 *μ*g) were cotransfected into skeletal muscle progenitor cells (Section 2.5.1) using lipofectamine stem as transfection reagent according to the manufacturer's manual. After selection for transfected cells with the addition of 2 *μ*g/ml puromycin, emerging single-cell colonies were transferred to 96-well plates for further expansion. Genomic DNA was prepared by isopropanol precipitation, and successful HDR-mediated mutation correction was assessed by amplification of an exon 4 sequence of CAPN3 using the PCR primers (5′-TGGGTCACTTTGTTCCTCCG-3′) and (5′-GGCTGGATTTTGCAACCCAC-3′) and consecutive sequencing to distinguish wildtype from mutant CAPN3.

#### 2.4.3. sgRNA Off-Target Analysis

To evaluate potential sgRNA off-target effects of the off-target prediction module from the CCTop-CRISPR/Cas9 target online predictor program [28] (https://cctop.cos.uni-heidelberg.de/.), using the default settings for the max, total mismatches, core length, and max core mismatches were applied.

### 2.5. In Vitro Differentiation into Skeletal Muscle Cells

Skeletal muscle differentiation in a two-dimensional culture system was performed according to the protocol of Chal et al. [29, 30]. Briefly, 0.9 × 10^5^ iPSCs were plated in one well of a 12-well plate with ROCK inhibitor. Media was changed every day until the density was around 20%. Differentiation media and growth factor sequential applications were adapted from Chal et al. [30]. At day 23, skeletal muscle morphology is clearly visible, and cells were used for FACS isolation of myogenic progenitors.

#### 2.5.1. FACS Isolation of Myogenic Progenitor Cells

Skeletal muscle cells differentiated in the two-dimensional culture system were dissociated using TrypLe (Thermo Fisher Scientific, 12563011) while mechanic dissociation took place every 15 minutes. After 45 min, dissociation was stopped, and single-cell suspension was filtered through a 40 *μ*m cell strainer. The cell suspension was stained with PE-anti-human CD82 antibody (BioLegend, 342103) in 2%BSA and 2 mM EDTA and sorted using FACS sorter (Beckman Coulter, MoFlo Astrios Cellsorter). For gating, unstained single cells from the 2D differentiated skeletal muscle cultures were used as a baseline control and excluded autofluorescence. FACS data were captured using Summit 6.3.1 and processed using Kaluza Analysis 2.1. from Beckman Coulter Inc.

#### 2.5.2. scRNA-Seq Analysis for Myogenic Progenitor Cell Markers

As a reference dataset, the GSE149451 dataset [31] was used, which profiles skeletal muscle progenitor/satellite-like cells differentiated according to the protocol of Chal et al. [29, 30]. For Seurat-based normalization, the SCT-transform approach was followed (https://satijalab.org/seurat/; [32, 33]). During the Seurat pipeline the sequencing depth, proportions of mitochondrial transcripts, cell cycle effects, and genes associated to stress [34] were regressed out.

#### 2.5.3. Immunohistochemistry for Myogenic Markers

Skeletal muscle differentiation cultures were stained after sorting CD82-positive cells after differentiation induction in the two-dimensional culture system as in 2.5. 10,000 cells were fixed onto objectives using THARMAC® Cellspin® I with 4% paraformaldehyde for 10 min. Cells were rehydrated with PBS and followed and permeabilized once with 0.1% (vol/vol) Triton-X100 in PBS. Subsequently, the cells were blocked with 5% BSA in PBS for 30 min at room temperature. Primary antibody incubation was performed for 1 h and secondary antibody incubation for 1 h, both at room temperature: primary antibody: anti-PAX7 (DHSB, 1 : 250); secondary antibody: goat anti-mouse IgG (H+L) cross-adsorbed secondary antibody, Alexa Fluor™ 488 (Thermo Fisher Scientific, 1 : 1000). Images were acquired on a Zeiss LAM 800. Images were processed using Zen Lite Blue version 4.0.3.

### 2.6. Electrophysiology

Further expansion of LGMD2A iPSC, LGMD2A isogenic iPSC, and WT (CB) iPSC-derived skeletal muscle fibers for electrophysiological evaluation was performed in skeletal muscle growth medium (PromoCell C-23060) after initial differentiation according to Mavrommatis et al. [35].

#### 2.6.1. Current Measurement

Membrane currents were measured at ambient temperature (22-24°C) using standard whole-cell patch clamp software ISO2 (MFK, Niedernhausen, Germany). Skeletal muscle cells were voltage-clamped at a holding potential of –90 mV, i.e., negative to EnAChR, resulting in inward Na+ currents. Every 10 s, voltage ramps (duration 500 ms) from -120 mV to +60 mV were applied to assess stability of the recording conditions and to generate I/V curves (membrane currents in response to depolarizing voltage ramps are shown as downward deflections). Signals were filtered (corner frequency, 1 KHz), digitally sampled at 1 KHz, and stored on a computer equipped with the hardware/software package ISO2 for voltage control, data acquisition, and data analysis. Rapid exposure to a solution containing acetylcholine was performed by means of a custom-made solenoid-operated flow system permitting a change of solution around an individual cell with a halftime of about 100 ms. For measurements, cells devoid of contact with neighboring cells were selected.

#### 2.6.2. Fluorescence Microscopy and Imaging

To monitor changes in [Ca2+]i, skeletal muscle cells were transiently transfected with pcDNA3[Twitch-2B] (Addgene, 49531) (0.25 *μ*g per 35 mm culture-dish) [36]. Skeletal muscle cells were transfected using either polyethyleneimine (PEI) or lipofectamine (Invitrogen) according to the manufacturer's instructions. Prior to experiments, cells were seeded on sterile, poly-L-lysine-coated glass coverslips and analyzed 48 h after transfections. All experiments were performed using single cells at ambient temperature. Fluorescence was recorded buckusing an inverted microscope (Zeiss Axiovert 200, Carl Zeiss AG, Göttingen, Germany) equipped with a Zeiss oil immersion objective (100x/1.4), a Polychrome V illumination source and a photodiode-based dual emission photometry system suitable for CFP/YFP-FRET (FEI Munich GmbH, Germany). For FRET measurements, single cells were excited at 435 nm wavelength with light pulses of variable duration (20 ms to 50 ms; frequency: 5 Hz) to minimize photo-bleaching. Corresponding emitted fluorescence from CFP (F480 or FCFP) or from YFP (F535 or FYFP) was acquired simultaneously, and FRET was defined as the ratio FYFP/FCFP. Fluorescent signals were recorded and digitized using a commercial hardware/software package (EPC10 amplifier with an integrated D/A board and Patch-master software, HEKA, HEKA Elektronik, Germany). The individual FRET traces were normalized to the initial ratio value before agonist application (FRET/FRET0).

#### 2.6.3. Solutions and Chemicals

For FRET measurements, an extracellular solution of the following composition was used (mmol/L): NaCl 137; KCl 5.4; CaCl2 2; MgCl2 1.0; HEPES/NaOH 10.0, pH 7.4. For whole-cell measurements of membrane currents, an extracellular solution of the following composition was used (in mmol/L): NaCl 120; KCl 20; CaCl2 0.5; MgCl2 1.0; HEPES/NaOH 10.0, pH 7.4. The pipette solution contained (in mmol/L) K-aspartate 100; KCl 40; NaCl 5.0; MgCl2 2.0; Na2ATP 5.0; BAPTA 5.0; GTP 0.025; HEPES/KOH 20.0, pH 7.4. Standard chemicals were from Merck. EGTA, HEPES, Na2ATP, GTP, and acetylcholine chloride were from Sigma-Aldrich.

### 2.7. Calpain-3 Western Blot Analysis

Skeletal muscle cells differentiated, as outlined in Section 2.6, from LGMD2A iPSC, their isogenic control, WT (CB) iPSC, and muscle biopsy were solubilized in lysis buffer (4 M urea, 20 mM Tris-HCL (pH 8,5), DTT, MgCl_2_, and 1% Triton-X-100) and further analyzed in samples of 5-8 *μ*g total protein/well by polyacrylamide gel electrophoresis and Coomassie brilliant blue (CBB) staining as described [37]. Proteins were transferred to a nitrocellulose membrane (Cytiva AmershamTM ProtranTM NC) for immunoblotting by semidry blot. The primary calpain-3 antibody (rabbit polyclonal, ab223766, Abcam Inc.) was 1 : 500 diluted in PBS-T (1xPBS, 0.05% Tween-20) with 3% BSA and the blot exposed at 4°C overnight. The secondary antibody POD goat *α* rabbit IgG (Sigma Inc.) was diluted 1 : 10.000 in 0.05% PBS-T with 3% BSA and exposed at room temperature for one hour. The chemiluminescent substrate (Radiance Q, Azure Biosystems, Dublin, CA, USA) was applied, and images were captured in an Azure 600 western blot imager (Azure Biosystems Inc.).

## 3. Results

For our study, we recruited, at the time of the biopsy, a 45-year-old male patient who showed a flaccid, atrophic, and proximally pronounced tetraparesis and was only able to stand unsteadily with support. His first symptoms began at the age of 12 years with distal contractures of the lower extremities, left-sided toe walking, and weakness of the pelvic girdle muscles. A degenerative myopathy was diagnosed histopathologically at the age of 16 years. Blood creatine kinase (CK) levels were moderately elevated (2- to 10-fold of the upper normal limit) during the disease course. At the same age, neurological examination showed a slight asymmetry of a flaccid, atrophic, proximally pronounced tetraparesis, bilateral scapula alata, and bilateral Trendelenburg and Gower signs matching clinical characterizations, according to Gallardo et al. [38]. The loss of the ability to climb stairs occurred at the age of 27 years, and the loss of the ability to get up from a chair occurred around the age of 33 years. Since the age of 40, a power wheelchair for mobility has been used. The patient was molecular genetically diagnosed at the age of 33 by sequencing all 24 exons and exon-intron transitions of his calpain-3-gene compound heterozygous for a Trp(TGG)130 point mutation towards Cys(TGC) in exon3 and the deletion of nucleotide A550 in exon 4. The deletion of A550 is the most common mutation of the calpain-3 gene in Europe [39] which results in a prematurely stopping of the protein synthesis. The change towards cysteine from the exon3 mutation most likely changes the protein conformation of the full-length protein. This W130C mutation has not been reported according to our literature research or been deposited in the Leiden Muscular Dystrophy pages© (https://www.dmd.nl/) [4].

Genome editing approaches using CRISPR/Cas9 [26, 40, 41] have been further developed by distinguishing successful biallelic events through the detection of fluorescent protein incorporation (GFP and RFP) into the genome [42, 43]. To these approaches, biallelic events can be distinguished by the presence of double GFP/RFP positive cells. For a more stringent selection process, we have developed a system that in addition to fluorescent interrogation, the doxycycline-inducible Tet-on system [44] aids in selecting for cells with successful bi-allelic events (Figure 1(b)).

### 3.1. Biallelic Genome Editing at the Calpain-3 Locus in LGMD2A Patient-Induced Pluripotent Stem Cells

**Figure 2:**
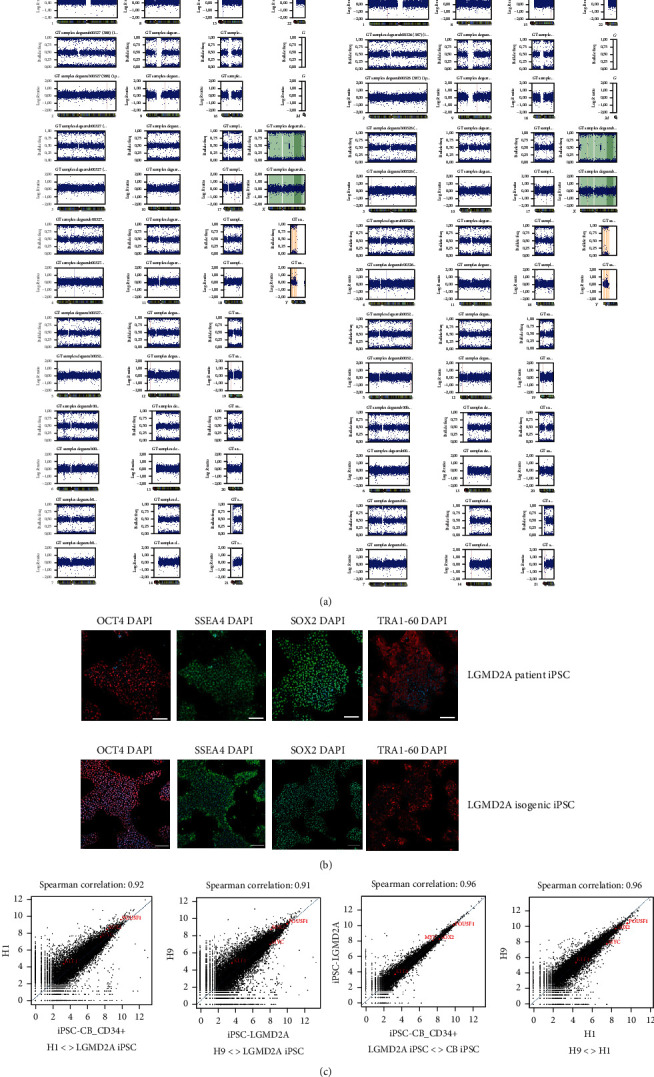
(a) Karyograms from patient and isogenic LGMD2A lines indicating no genomic alterations occurrence during genome editing. (b) Immunocytochemistry for the pluripotency markers Oct4, Sox2, SSEA4, and TRA1-60 displaying the pluripotent state of the LGMD2A iPSC as well as their isogenic control iPSC after genome editing. Scale bars 100 *μ*M. (c) Scatter plots comparing global gene expression profiles between H1 human ESC and LGMD2A iPSC, H9 human ESC and LGMD2A iPSC, CB iPSC, and LGMD2A iPSC, and H1 and H9 human ES cells (from left to right) and the corresponding Spearman correlation. H1 and H9 human ESC for analyses were acquired from the GSE73211 data set.

**Figure 3:**
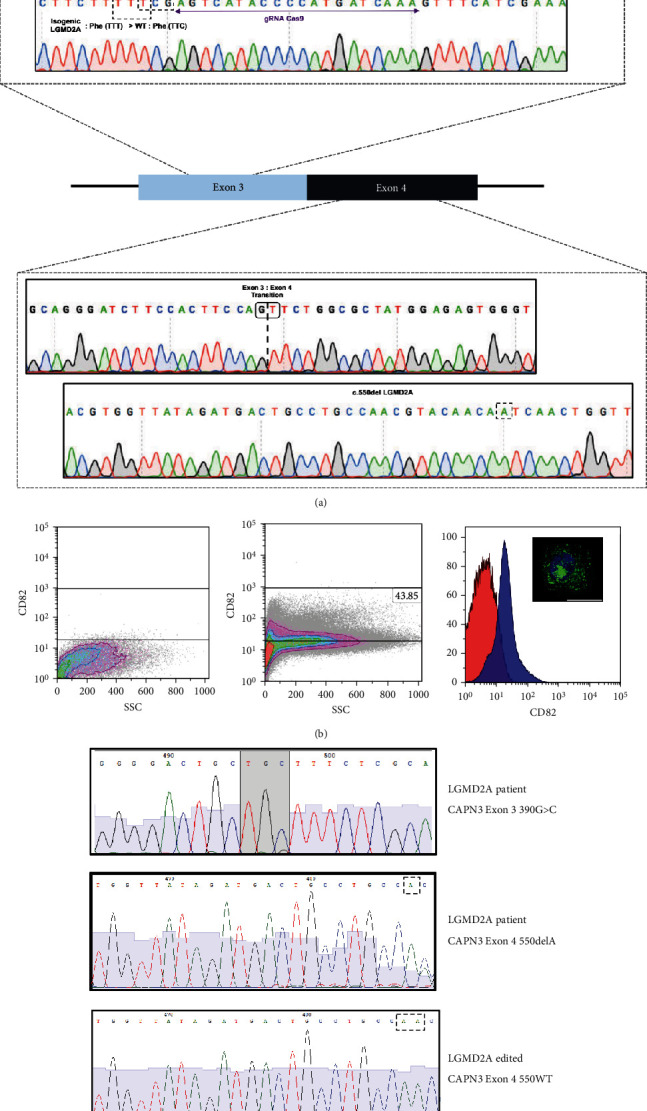
(a) Scheme displaying the new EXON3/4 CAPN3 conformation together with pyrograms emphasizing all silent introduced mutations TTT:TTC (Phe), CTG:CTT (Leu), EXON3 to EXON4 transition, as well as rescues in EXON3: 130Cys(TGC-LGMD2A) > Trp(TGG-WT) and EXON4: c.550delA. (b) CD82+ FACS sorting of skeletal muscle progenitor cells differentiated from LGMD2AiPSC: (i) unstained skeletal muscle population (left), (ii) differentiated and stained skeletal muscle cells with 43.85% gated myogenic progenitors/satellite-like cells (middle), and (iii) visualization of the shift of unstained and stained populations (red: unstained control; blue: CD82 stained cells) (right) and PAX7 immunohistochemistry of CD82+ progenitor cells after plating via cytospin. Pax7 (green) and DAPI (blue). Scale bar: 20 *μ*m (right). (c) DNA sequencing electropherograms from genomic DNA of LGMD2A iPSC differentiated muscle cells at the exon3 390G > C CAPN3 locus (upper panel), at the exon4 550delA CAPN3 locus (middle panel), and after CRISPR/Cas9-gene-correction of the exon4 550delA CAPN3 locus (lower panel).

**Figure 4:**
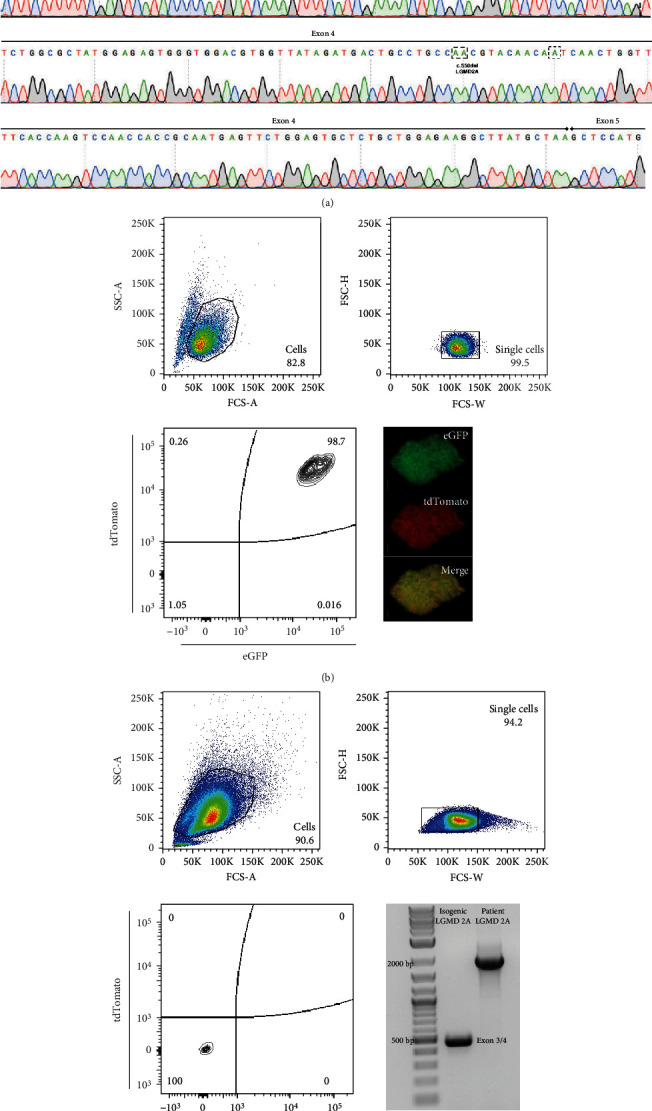
(a) Sequencing diagram of amplified calpain-3 cDNA after genome editing, proves precise fusion between exon3 and exon4 and the restoration to WT CAPN3 sequence. (b) FACS gating strategy to distinguish/acquire cells with bi-allelic modifications (tdTomato and eGFP double-positive population). Lower left panel: immunofluorescence picture of an iPSC colony after the double-positive sorting. (c) FACS gating strategy to distinguish/acquire cells with t removal of the selection cassette (tdTomato and eGFP double-negative population). Lower left panel: PCR of verification of cassette excision at CAPN3 locus and chimeric Exon3/4 generation on the level of genomic DNA (lane “Isogenic LGMD2A”; PCR control lane “patient LGMD2A”).

In our attempt to model the limb-girdle muscular dystrophy type 2A (LGMD2A) with induced pluripotent stem cell-derived skeletal muscle cells and excluding at the same time any variance arising from the cell line genetic background, we intended to generate a hiPSC isogenic line from our LGMD2A patient-derived iPSC line (Figures 1(a) and 2). In our case, the patient line was harboring two mutations, one-point mutation at exon3 (c.390: G > C) and one base deletion (c.550delA) at exon4, responsible for amino acid replacement and premature termination codon generation, respectively (Figures 1(a), 1(b), and 3(c)). Based on existing protocols [42, 43] for performing biallelic modifications in human iPSC lines in a single step, we thought of developing a strategy, which would allow us to discriminate the cells with successful bi-allelic modifications, whereas at the same time, a new functional chimeric exon 3-4 by skipping the intermediate intron can be generated (Figures 1(b) and 3(a)). For distinguishing all the biallelic events, we thought of generating a dependent loop between two selection cassettes: one selection cassette is expressing the Tet trans-activator under a constitutive promoter, while the other is expressing a fluorescent protein (tdTomato-red) and a selection drug (puromycin) under the Tet-CMV conditional promoter (Figure 1(b)). Both cassettes harbor a constitutively expressed green fluorescent protein (eGFP). This system allows red/green-visualization of cells after successful biallelic modification and further enrichment after administration of puromycin and purification via FACS sorting to full purity (Figure 4(b)). For the removal of both cassettes, ITR sequences flanked the selection cassettes, which allows piggyBac transposase mediated removal (Figure 4(c)). Within a few FACS-sorting experiments, double-positive and, in the second step, after piggyBac transposase transfection, double-negative cell populations could be identified. Following excision, the depletion of the intermediated intron did not affect the integrity of Calpain3 mRNA (Figure 4(a)). In addition, karyotype integrity investigation did not reveal any alterations arising from the genome editing process, thereby verifying a functional isogenic line (Figure 2(a)). From the output sequences of an off-target effects prediction for the used 5′-TTTGATCATGGGGTATGACT-3′ sgRNA, we evaluated the first ten hits in probability by sequencing, where we could not detect any sequence alterations (Supplemental Table 1). Finally, Western blot analysis evaluating calpain-3 expression demonstrated the 94 kDa full-length calpain-3 protein band for the LGMD2A iPSC and their isogenic counterparts similar to WT iPSC-derived skeletal muscle cells (Supplemental Figure 1).

### 3.2. Genome Editing at the Calpain-3 Locus in LGMD2A Patient-Derived Skeletal Muscle Progenitor Cells

In our attempt to provide a protocol to allow genome editing not on the level of iPSC but on LGMD2A patient skeletal muscle progenitor cells, we decided to evaluate, if CRISPR/Cas9 genome editing was feasible on CD82+ progenitor from human iPSC differentiated in a 2D cell culture protocol [29, 30]. Several groups have described surface marker combinations for human skeletal muscle progenitor/satellite cells, among the most prominent CD82+ and CXCR4+/CD56+/CD29+ combinations [45–47]. FACS can be used to isolate homogenous subpopulations from heterogenous populations, e.g., from biopsies or in vitro differentiated cells. We differentiated the LGMD2A iPSCs for 23 days and used FACS to sort a myogenic progenitor population that separated from myoblasts and myocytes. Therefore, we used CD82-marking as previously described [45, 48]. On day 23, after differentiation induction, LGMD2A-derived cells presented with 44% CD82-positive cells in the culture (Figure 3(b)), which could be plated as a monolayer culture. In a control experiment LGMD2A-isogenic iPSC, generated with the biallelic genome editing above, derived progenitors presented with 49% CD82-positive cells on day 23 of differentiation. The plated CD82-positive populations showed positivity for the skeletal muscle progenitor/satellite cell marker Pax7 (Figure 3(b), Supplemental Figure 2). Further FACS analysis of isolated and cultured myogenic progenitors/satellite-like cells indicated a downregulation of CD56 as long as they are cultivated as single cells (data not shown). About 125.000 cells were diluted in a dilution series until about 0.5 cells/100 *u*l was achieved and then transferred to 96 wells of a 96-well plate. From this plate, ten colonies were further expanded and propagated. From all ten cell lines, genomic DNA was isolated, and the calpain-3 locus exon 3 and exon 4 areas were amplified by PCR. Two cell lines out of ten presented with a rescue of the exon 4 LGMD2A mutation to the wild type (Figure 3(d)), which corresponds to successful homology-directed repair in 20% of the clones. In contrast from 96 cell lines expanded after adding the exon 3 W130C mutation correcting targeting vector and ssODNs, rescue of the exon 3 mutation could not be verified by sequencing in any of the lines.

### 3.3. Skeletal Muscle Differentiation and Electrophysiological Characterization of LGMD2A iPSC and Their Isogenic Control iPSC Lines

**Figure 5:**
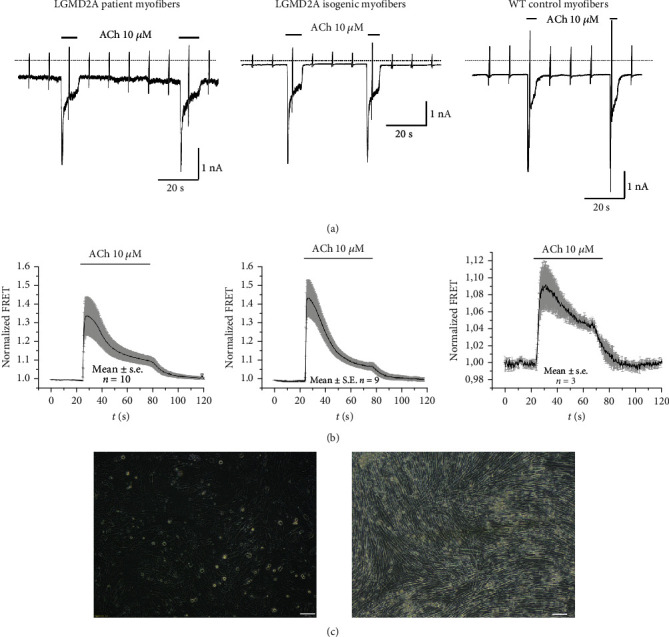
(a) Representative recordings (*n* = 8 cells) of ACh-induced currents in LGMD2A patient, their isogenic control, and WT (cord blood) iPSC-derived skeletal muscle cells. ACh (10 *μ*M) was applied as indicated by the bar. Holding potential -90 mV. Downward deflections represent membrane currents in response to depolarizing voltage ramps (duration 500 ms) from -120 mV to +60 mV. Dashed line indicates zero current level. (b) Summarized FRET recordings in LGMD2A iPSC, their isogenic control, and WT (cord blood) iPSC-derived skeletal muscle cells; transfected with Twitch2B to monitor the increase in [Ca2+]i during ACh application. (c) Morphology of LGMD2A iPSC derived skeletal muscle cells on day 12 and day 40 after differentiation induction (2D protocol) scale bars 100 *μ*M.

We successfully patterned myofibers from the LGMD2A iPS cells and investigated the physiological properties (Figure 5). To probe functionality of nACHRs in LGMD2A-patient skeletal muscle-derived cells, we measured ACh-induced currents in the whole-cell configuration. As demonstrated by the representative current recording, cells from LGMD2A patients were responsive to acetylcholine similar to time-matched LGMD2A isogenic cells and WT iPS cells, which served as controls. However, in cells from LGMD2A patients, we frequently observed current fluctuations and a slight shift of the holding current, probably due to instability of the cell membrane related to the LGMD2A phenotype (Figure 5(a), left panel). Thereby, our patient and corresponding isogenic control lines provide the basis for further LGMD2A iPSC-based muscular dystrophy modelling on the physiological level.

## 4. Discussion

Human iPSCs have been generated and CRISPR/Cas9 genome edited from LGMD2B and 2D patients caused by mutations in the dysferlin and alpha-sarcoglycan genes [49] as well as three LGMD2A patients caused by mutations in exons 17, 22, and 24 of the calpain-3 gene [50].

Overall, we are providing LGMD2A iPSC of a compound heterozygous patient presenting a new combination of calpain mutations in exons 3 and 4, matching isogenic control iPSC generated with a new selection strategy as well as an outline of how to further perform CRISPR/Cas9 genome editing on the level of skeletal muscle progenitor cells.

Genome editing has been used to introduce sequence-specific alterations in the genome of cells to derive gene-knockout cell lines or create specific mutations for various purposes [20]. Most current approaches use the CRISPR/Cas9 nuclease system, which was originally discovered as an RNA-guided endonuclease bacterial immune response to foreign DNA [40]. It was further developed to allow double-strand breaks in specific regions of mammalian genomes [26, 41]. The applications of CAS nucleases have increased the frequency of double-strand breaks at specific genomic sites in human pluripotent stem cells above 1% and higher. When combined with dual fluorescence selection strategies biallelic genome modifications can be achieved with high recombination frequencies [42, 43].

Mandal et al. have extended the application of CRISPR/Cas9 to directly knock out genes in human CD34+ blood stem/progenitor cells [51]. We further expanded the utility of these genome editing strategies to target neural progenitor cells (NPCs) by demonstrating efficient homology-directed repair at the TAU locus in NPCs differentiated from frontotemporal dementia (FTD) patient iPSCs [20, 52]. Significant homologous recombination frequencies were achieved in NPCs after CRISPR/Cas9 system application (12% (3 edited/25 clones) in line with the reported high CRISPR/Cas9-directed knockout frequencies (27%) in human CD34+ stem/progenitor cells [51, 52].

In this report, we are extending our investigations on CRISPR/Cas9 genome editing to iPSC-derived skeletal muscle cells. In principle, genome editing of iPS cell-derived cells can be efficiently undertaken (i) on the level of pluripotent stem cells but also on (ii) progenitor cells derived therefrom (Figure 1).

To apply these concepts to our LGMD2A patient biopsy, we were confronted with the specific genetic background of our patient line with two mutations in exon 3 and exon 4 at the calpain 3 locus (W130C, 550delA). To overcome the obstacle of performing genome editing into two different exons in a single step, we thought of generating an additional deletion by excising the intermediate intron during the homology-directed repair (HDR) pathway, since the ORF of CAPN3 gene seemed to follow the GT-AG rule regarding intron-exon boundaries (Figure 1(b)). Another challenge was that the genome modification ought to be biallelic, since LGMD2A is an autosomal recessive disease and our patient line was compound heterozygous. Therefore, to distinguish these biallelic events, we came up with the idea of generating a dependent loop between the donor templates that would direct the selection of biallelic events only. To our knowledge, our report is a first-time description of CRISPR/Cas9 genome editing including deletion, insertion, and base exchange in a single step (Figure 3(a)). The genomic integrity is still preserved (Figure 2).

Based on concepts as outlined by Arias-Fuenzalida et al. [42] and Eggenschwiler et al. [43] for performing biallelic modifications in human iPSC lines in a single step, the overall design of our approach avoids laborious quantifications for genome editing. The approach is efficient independently, since successfully biallelic edited clones are selected and expanded in a two-step strategy. For that reason, first, we introduced a reporter GFP/RFP system to target and isolate via FACS biallelic modifications, and second, we introduce an inducible (TRE-doxycyclin) puromycin resistance cassette to select successful biallelic events, which are further depended on the presence of both plasmids within the same cell. Similar to when we excise the cassette, the absence of the dual reporter system on edited cells aids us to identify and obtain genome-edited cells via FACS sorting.

A potential drawback of our approach is that potential regulatory sequences or alternative splicing signals in the intron between exon 3 and exon 4 might be omitted after editing. The calpain-3 bands within the Western blot analysis present the same between LGMD2A iPSC, their isogenic line, and further controls (Supplemental Figure 2). The compound heterozygosity of our patient distinguishes oneself with a full-length calpain-3 protein from the allele with the exon3 point mutation most likely with an altered conformation. Thereby, an equal banding can also be expected between these patients' iPSC-derived skeletal and their isogenic counterparts as well as control cells. The observance of a normal amount of calpain-3 protein when using Western blotting has been studied in a cohort of 58 LGMD2A patients, whereby about 20% had normal calpain-3 protein amounts [53].

For our second editing approach, we are targeting myogenic progenitor cells which are suggested to be profiled by certain genetic and surface molecule marker combinations [29, 54–58]. FACS-selection by combination of surface markers like CD56 or CD82 has been introduced by various groups, whereby CD82+ presents as a single myogenic progenitor marker [45–47]. We are providing an in silico scRNAseq analysis that shows that human CD82+ progenitor cells differentiated from iPSC, according to Chal et al. [29], also coexpress the satellite cell markers Pax7, Myf5, and MyoD1 (Supplemental Figure1).

We were able to edit the exon 4 550delA mutation in these CD82+ sorted progenitor populations with a frequency of 20%. In contrast, the editing of the exon 3 W130C mutation was not successful in 96 lines expanded. In our previous CRISPR/Cas9 experiences with genome editing at the TAU-locus in neural progenitor cells, we had achieved a frequency of 12% [52]. We can just speculate that some genomic sides are more refractory to the CRISPR/Cas9-mediated cut or more resistant to the following HDR repair process, resulting in the different frequencies of successful editing. In our LGMD2A patient, we had a compound heterozygosity concerning the two mutations in exon 3 and exon 4, so that the locus on the allele with the exon 4 mutation might have been more open, permissive for the editing via the single-stranded DNA oligonucleotide approach then the other allele with the exon 3 mutation.

Both of our approaches on the level of LGMD iPSC as well as progenitor cells allowed HDR in reasonable frequencies to rescue in Europe's most common calpain-3 exon 4 550delA mutation. Our first approach of targeting on the level of LGMD iPSC cannot really be envisioned for human cellular therapies given the complexity of the process to generate autologous patient iPSC and the further editing in a GMP- (good manufacturing practice-) compliant process. We are presenting this new LGMD2A iPSC line and its isogenic control primarily as a resource for further disease modelling.

The isolation of myogenic progenitor cells based on surface marker from patient material is in rapid progress [45–47, 59]. Current studies expand our knowledge of how these progenitor/satellite-like cells might be expanded in vitro [29, 31, 35]. We envision a process, where these precursor populations are FACS-isolated from patient cells, expanded, briefly edited within a short culture period using episomal CRISPR/Cas9 editing plasmids and ssODNs, and then infused back to repopulate a certain skeletal muscle area. Complementary to the currently discussed translational applications of the CRISPR/Cas9 system for muscular dystrophies [60], we are adding here one successful example of editing in LGMD2A patient-derived skeletal muscle progenitor cells. CD82/Pax7-positive target populations might have translational potential for CRISPR/Cas9-mediated editing in the context of muscular dystrophies, insofar as HDR in differentiated skeletal muscle remains challenging.

## 5. Conclusion

Here, we successfully generated induced pluripotent cells from a patient with limb-girdle muscular dystrophy and devise two strategies to rescue the disease-specific genomic mutations: (i) on the level of LGMD2A-iPSC, we have combined CRISPR/Cas9 genome targeting with a FACS and Tet transactivator based biallelic selection strategy which resulted in a new functional chimeric exons 3-4 without the two CAPN3 mutations. (ii) On the level of LGMD2A-iPSC-derived CD82+/Pax7+ skeletal muscle progenitor cells, we demonstrate CRISPR/Cas9 mediated rescue of a highly prevalent exon 4 CAPN3 mutation. The first strategy specifically provides isogenic LGMD 2A/R1 corrected iPSC for disease modelling, and the second strategy can be further elaborated for potential translational approaches.

## Data Availability

RNA sequencing datasets produced in this study are deposited in the Gene Expression Omnibus (GEO) under accession code GSE147513 (https://www.ncbi.nlm.nih.gov/geo/query/acc.cgi?acc=GSE147513).

## Supplementary Materials

Supplementary 1 Supplemental Figure 1: western-blot analysis of LGMD2A, LGMD2A isogenic, and WT iPSC-derived skeletal muscle cells as well as muscle biopsies depicting the 94 kDa calpain-3 band and its degradation products. Lower part of the figure: Coomassie staining before blotting.

Supplementary 2 Supplemental Figure 2: UMAP feature plots predicting relative expression of the surface marker CD82, the satellite cell marker PAX7, MYF5, and MYOD1, and the myogenic markers MYOG and MYH3 of skeletal muscle progenitor cells differentiated from human iPSC according to the two-dimensional differentiation protocol used for genome editing of CD82+ progenitor cells [29, 31].

Supplementary 3 Supplemental Table 1: predicted off-target effects of the 5′-TTTGATCATGGGGTATGACT-3′ sgRNA sequence using ccTOP CRISPR/Cas9 target online predictor program and primers used to amplify the genomic region before sequencing.
