# Supplementary figures and images for: CRISPR/Cas9 Genome Editing in LGMD2A/R1 Patient-Derived Induced Pluripotent Stem and Skeletal Muscle Progenitor Cells
Source: Stem Cells Int. 2023 Nov 9;2023:9246825. doi: 10.1155/2023/9246825 (PMC10653971; doi:10.1155/2023/9246825)

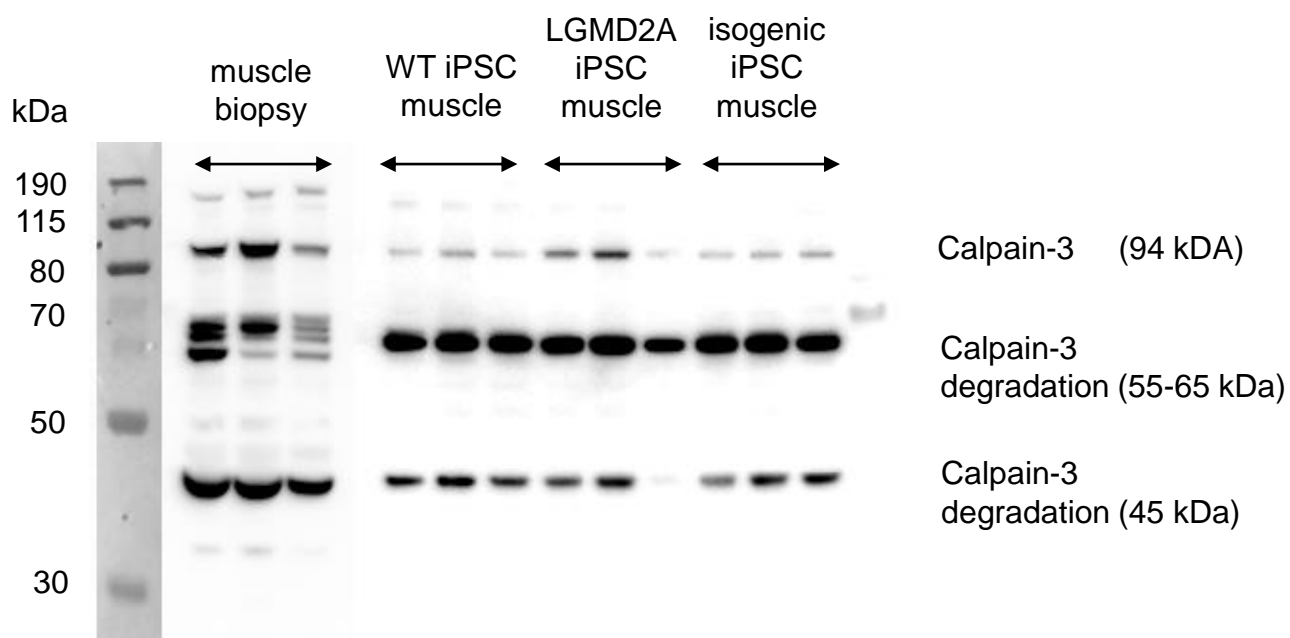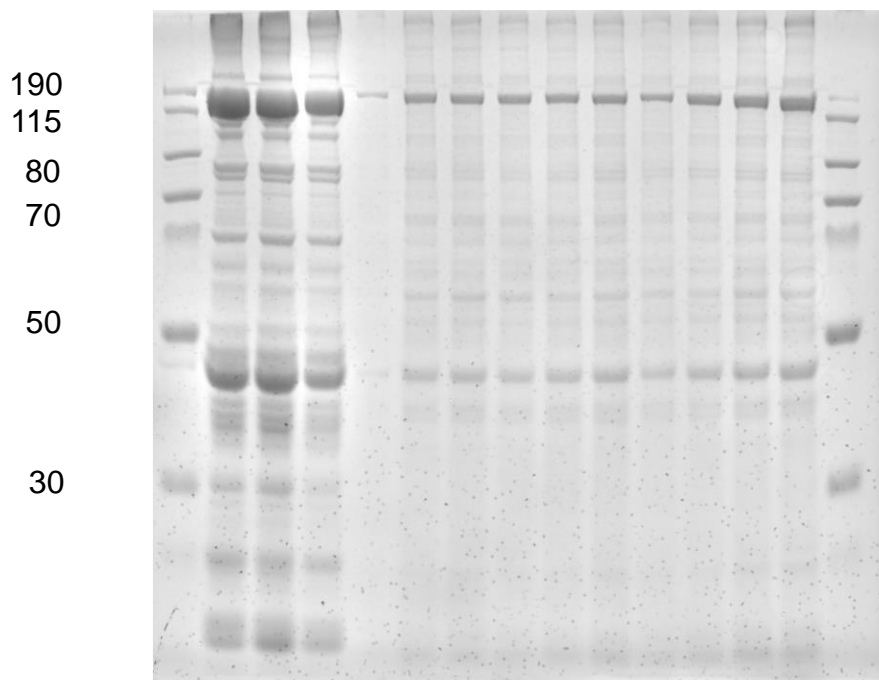

**Supplemental Figure 1**

Supplement: Supplementary 1 — Supplemental Figure 1: western-blot analysis of LGMD2A, LGMD2A isogenic, and WT iPSC-derived skeletal muscle cells as well as muscle biopsies depicting the 94 kDa calpain-3 band and its degradation products. Lower part of the figure: Coomassie staining before blotting. [file 9246825.f1.pdf]

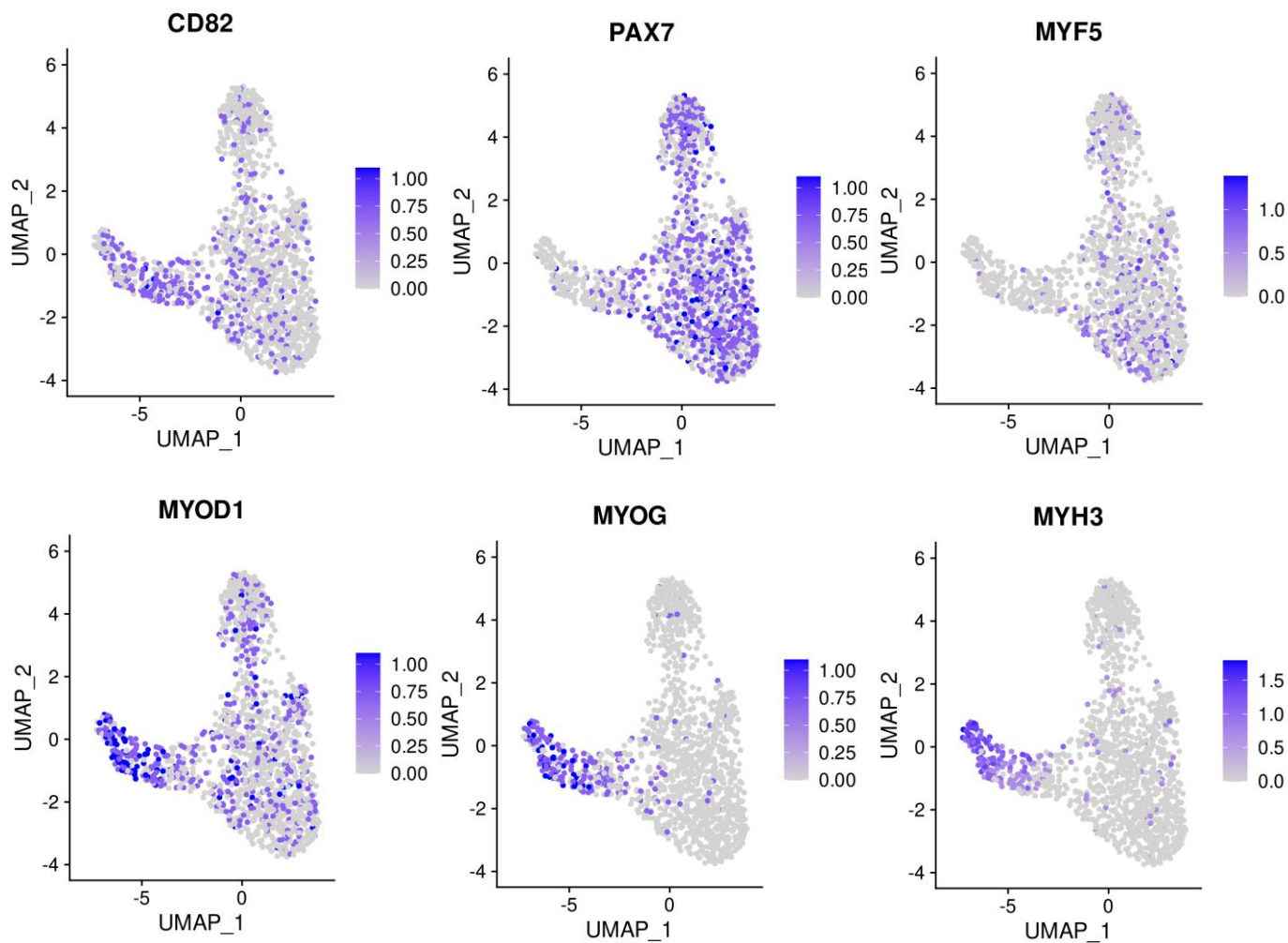

**Supplemental Figure 2**

Supplement: Supplementary 2 — Supplemental Figure 2: UMAP feature plots predicting relative expression of the surface marker CD82, the satellite cell marker PAX7, MYF5, and MYOD1, and the myogenic markers MYOG and MYH3 of skeletal muscle progenitor cells differentiated from human iPSC according to the two-dimensional differentiation protocol used for genome editing of CD82+ progenitor cells [29, 31]. [file 9246825.f2.pdf]
