# Supplementary material for: CRISPR/Cas9 Genome Editing in LGMD2A/R1 Patient-Derived Induced Pluripotent Stem and Skeletal Muscle Progenitor Cells
Source: Stem Cells Int. 2023 Nov 9;2023:9246825. doi: 10.1155/2023/9246825 (PMC10653971; doi:10.1155/2023/9246825)
Supplement: Supplementary 3 — Supplemental Table 1: predicted off-target effects of the 5′-TTTGATCATGGGGTATGACT-3′ sgRNA sequence using ccTOP CRISPR/Cas9 target online predictor program and primers used to amplify the genomic region before sequencing. [file 9246825.f3.pdf]

Off -Target Site	Off-Target Sequence	PAM	Strand	MM	Forward detection Primer	Reverse detection Primer	Gene	Position
**Chromosome 15: 42386225 - 42386247**	TTTGATCA [TGGGGTATGACT]	CGG	-	0	TTCTCCTTCCCTGGGTTGAC	CTCGGCTGGATTTTGCAACC	CAPN3	Exonic
**Chromosome 5: 125465023 -125465045**	CTGAGTCA [TGGGGTATGACT]	AGG	-	4	CCCCAAGGAGTGCCAGAGTAA	TGGAGAAGCACTACCTTATGGC	RP11-756H20.1	Intergenic
**Chromosome 14: 66857796 - 66857818**	GTTGCTGC [TGGGGTATGACT]	GGG	-	4	CCTCTTCCTAAGCGAAACTGA	GTGGTTCCCCTCTGTGTAGGG	GPHN	Intronic
**Chromosome 2: 167762690 - 167762712**	TTAGAGTT [TGGGGTATGACT]	GGG	+	4	AGCAGGCCACTACTACCTCT	CAGGTCTATTCACCCTCTGGG	CTAGE14P	Intergenic
**Chromosome 5: 33894317 - 33894339**	GTGGATCA [AAGGGTATGACT]	GGG	-	4	GTATTCTGCCAGCCCTTCCAA	TCTACGCTGAGGTAACTCCAAG	ADAMTS12	Intergenic
**Chromosome 6: 87520187 - 87520209**	GAAGATCA [TGGGATATGACT]	GGG	-	4	ACTAAGCCACTGAGAGCAGAT	TCCCTCCCTTGTAGAGCAGT	RARS2	Exonic
**Chromosome 14: 57973592 - 57973614**	TTGCATTA [TGGGATATGACT]	TGG	+	4	CTGTGGTTCTGTTTTCCTGCTG	GCAGTTTGGGCTTCAGGGAG	SLC35F4	Intronic
**Chromosome X: 75062360 - 75062382**	CTTCATCA [TAGAGTATGACT]	GGG	+	4	TGGAAAAAGGGGGATAGGCAT	TCTTCCCACCTGCTATGAAGT	ABCB7	Exonic
**Chromosome 11: 133745438 - 133745460**	TGTTCTCA [TGGGGAATGACT]	AGG	-	4	GGCAGGAGCAGGCAAGAATA	GTAGCAACCCAGGAGTACTGT	RP11-448P19.1	Intergenic
**Chromosome 10: 105967626 - 105967648**	TTTGATAT [AGAGGTATGACT]	TGG	+	4	AGTGGCCATGGGAGATTGTC	AGCACATAACGCATGCTCAG	N / A	Intergenic
**Chromosome 4: 41570773 - 41570795**	ATTGGCCA [TGGGGTGTGACT]	TGG	+	4	ATCCTGGTTTGGACCTGTGC	ACCCTAACGAACAGTCCTCCA	LIMCH1	Intronic

Supplemental Table 1
